# Allosteric pathway selection in templated assembly

**DOI:** 10.1126/sciadv.aaw3353

**Published:** 2019-10-11

**Authors:** Martijn van Galen, Ruben Higler, Joris Sprakel

**Affiliations:** Physical Chemistry and Soft Matter, Wageningen University and Research, Stippeneng 4, 6708 WE Wageningen, Netherlands.

## Abstract

Assembling large numbers of molecular building blocks into functional nanostructures is no trivial task. It relies on guiding building blocks through complex energy landscapes shaped by synergistic and antagonistic supramolecular interactions. In nature, the use of molecular templates is a potent strategy to navigate the process to the desired structure with high fidelity. Yet, nature’s templating strategy remains to be fully exploited in man-made nanomaterials. Designing effective template-guided self-assembling systems can only be realized through precise insight into how the chemical design of building blocks and the resulting balance of repulsive and attractive forces give rise to pathway selection and suppression of trapped states. We develop a minimal model to unravel the kinetic pathways and pathway selection of the templated assembly of molecular building blocks on a template. We show how allosteric activation of the associative interactions can suppress undesired solution-aggregation pathways and gives rise to a true template-assembly path.

## Teaser

Allostery plays a critical role in selecting high-fidelity assembly pathways.

## INTRODUCTION

Despite its vast complexity, nature is able to construct functional nanostructures from small biomolecular building blocks with an astonishing precision and fidelity. Virtually all functional biological structures that form the basis of life, including protein complexes, lipid bilayers, and DNA double helix, self-assemble spontaneously from precisely defined building blocks guided by well-balanced supramolecular interactions (*1*). It is remarkable that the self-assembly of complex structures in dense and crowded surroundings occurs with such a high degree of fidelity, as it requires a large number of molecular building blocks to come together in a precise order and orientation. High-precision molecular assembly is governed by a delicate balance between repulsive and attractive supramolecular forces, excited by thermal fluctuations, which prevent the formation of kinetically trapped states and structural polymorphism (*2*–*4*). Driven by thermal motion, self-assembly is a chaotic process consisting of numerous docking, folding, and reorganization steps, occurring in a complex energy landscape that is rich in local energy minima and activation barriers.

Nature has developed a number of strategies to navigate assembly processes through this complex landscape. One particularly potent strategy is the use of a molecular template as a blueprint to guide the assembly process. The use of a template can greatly enhance both the efficiency and the fidelity of the assembly process: The template can act as a molecular staging area, forcing the building blocks to assemble in specific morphologies that are otherwise not possible (*5*). Templates can also catalyze the assembly process by attracting building blocks, increasing their effective local concentration with respect to the bulk solution. A prominent example of templated assembly is the assembly of viral capsids, in which the organization of the coat proteins is guided by the nucleic acid polymer it will ultimately encapsulate (*6*, *7*). Other examples include the self-organization of light-sensitive rhodopsin proteins on retinal membranes (*8*, *9*) and septin assemblies on actin fibers, which control cytoskeletal structure and dynamics (*10*).

The effectiveness of templated assembly as a control mechanism in nature has also triggered the interest in using molecular templating to gain control over the assembly of molecular building blocks into synthetic nanostructures (*11*), such as in template-guided covalent and noncovalent polymerization (*12*, *13*); the use of carbon nanotubes or DNA origami to template the assembly of proteins, polymers, or nanoparticles (*14*–*16*); or the creation of bioengineered artificial viruses for gene delivery (*17*, *18*).

The final states of templated assembly processes have been studied in a variety of biological and synthetic systems, yet the underlying kinetic pathways are still poorly understood. Unraveling these pathways is crucial not only to understand how nature can create intricate and adaptable structures with high fidelity but also as a design principle to gain control over kinetic pathways to increase the fidelity with which synthetic nanostructure self-assembles. Several analytical models for the kinetic pathways of templated assembly have been proposed (*7*, *19*, *20*). A prominent example is the kinetic zipper model for the rod-shaped tobacco mosaic virus, in which the templating process is modeled as a thermodynamic process of monodirectional elongation that starts from a predefined nucleation site and the kinetics are steered by the interactions between the building blocks (*20*). Coarse-grained molecular dynamics simulations have also been used to follow templated assembly in time (*21*, *22*). Although both analytical approaches and coarse-grained models successfully describe the template-assembly kinetics, many describe specific cases of templated assembly, such as predesigned capsid shapes belonging to specific types of viruses. Unraveling the generic design rules of templated assembly will require a more generic model, capable of being tuned to predict a wide variety of template-assembling architectures to address the question that is central to this paper: Which design requirements must a system obey to efficiently and effectively guide a multitude of building blocks to a predesigned templated structure?

In this paper, we present a minimal simulation model to capture the essential and generic features of templated assembly in macromolecular systems. Our simulations reveal that assembly occurs along two primary kinetic pathways, occurring either through aggregation of building blocks in solution followed by template binding or by true templated assembly in which the template is the staging area for the subsequent organization of the building blocks into a functional structure. While the relative balance between these two pathways can be tuned by the chemical design of the system, the aggregation pathway remains important under all conditions if no further precautions are taken. This implies that without means of pathway selection, solution aggregation and subsequent kinetic trapping is an important pitfall in templating systems. We show how allosteric activation of lateral interactions can almost completely suppress the aggregation pathway and lead to full and true template-guided assembly. We combine our simulations with a kinetic reaction model to disentangle the complex process into a set of primary supramolecular reactions. This predictive and minimal model allows a rational design of new templated assembly strategies to go toward high-fidelity structuring of synthetic nanomaterials using molecular blueprinting.

## RESULTS

**Fig. 1:**
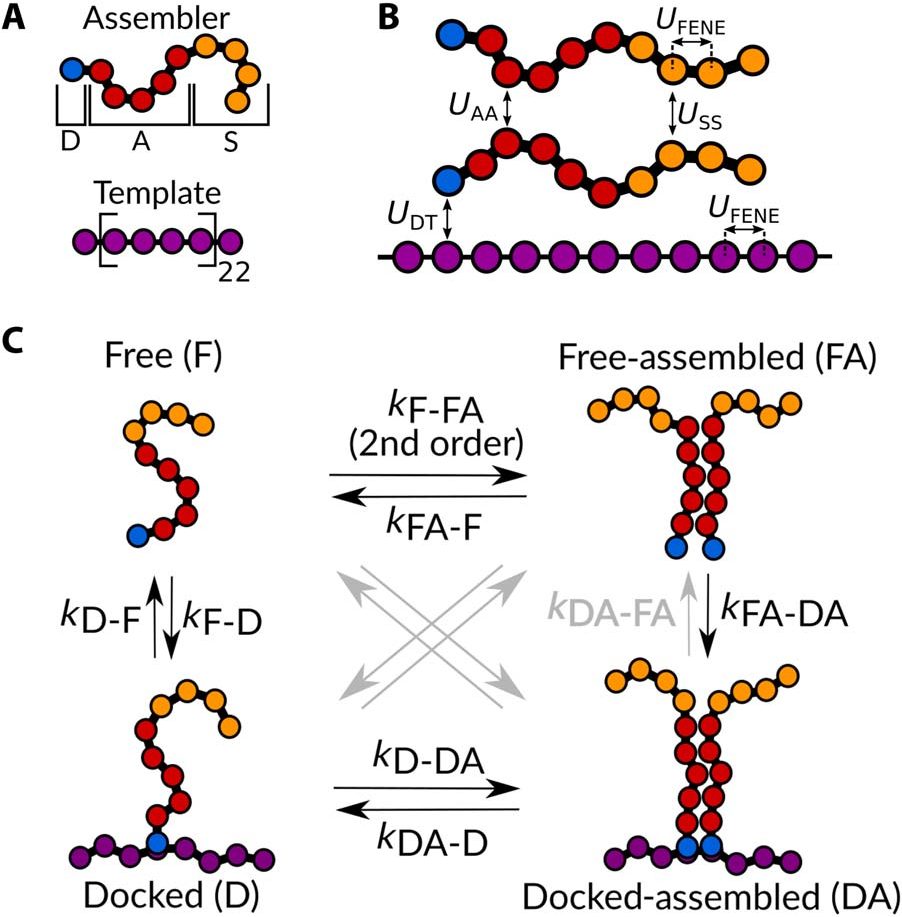
Overview of our template-assembling system and kinetic model. (**A**) Structure of assembler units and template as proposed in our model and used in the simulations. Assemblers consist of a part responsible for template binding, the docking domain (D), an assembly domain (A) capable of providing lateral attractions between assemblers, and a purely repulsive stability domain (S). We use a template consisting of 90 beads, *T*_90_, and assemblers made from one docking domain, five assembly domains, and four stability domains (*D*_1_*A*_5_*S*_4_). (**B**) Schematic overview of all pair interactions used in our simulations. Note that the remaining repulsive WCA potentials besides *U*_SS_ are not shown. (**C**) Suggested assembly states and pathways, including the rate constants defined in the kinetic model. Gray arrows indicate transitions that were ignored in our model.

In essence, templated assembly requires two primary ingredients (Fig. 1A). First, a multivalent template, such as a polymer chain with supramolecular binding capabilities, must be present onto which multiple biomolecular building blocks can bind. Second, we need numerous building blocks, here denoted as the “assemblers,” which bind this template and assemble into the final structure. These building blocks typically feature three domains: a domain to dock to the template (domain D), a domain to provide lateral associative interactions between template-bound molecules (domain A), and a stability domain (domain S) that provides a means to moderate associative forces, solubilize the final structure, and regulate its size and shape. This functionality triad is found in many biological building blocks and various bioinspired systems (*7*, *12*, *17*). In our work, we model the assemblers as short flexible chains that feature these three domains in a consecutive fashion along the chain (Fig. 1A). The repulsive and attractive interactions between assemblers and between assembler and template are encoded by simple potentials, as outlined in Fig. 1B.

Within this minimal model, we can identify several possible states (Fig. 1C). Initially, assemblers are free in solution (state F) from which they have the possibility of diffusing toward a template and binding to form a docked state (state D). Attractive interactions between assemblers, mediated by their assembly domain, can lead to the association of assembles in solution in the absence of a template, leading to a freely diffusing assembled aggregate (state FA). Last, the scenario of interest is that lateral association of multiple assemblers is mediated by the binding and accumulation on the template, leading to the desired final product of a docked and assembled state (state DA).

The transition from one state to the next is governed by a complex set of reversible supramolecular reactions; even with two molecular species and four possible states, this gives rise to a vast number of kinetic pathways from the initially dissolved state to the final architecture. We can describe the dynamical transitions between the four states in this conceptual model as a supramolecular reaction network (Fig. 1C) that is governed by the following set of differential equations

$$\frac{d[F]}{dt}=-k_{\text{FFA}}{\left[F\right]}^{2}-k_{\text{FD}}[F]\cdot \xi +k_{\text{FAF}}[\text{FA}]+k_{\text{DF}}[D]$$ (1)

$$\frac{d[D]}{dt}=k_{\text{FD}}\cdot \xi [F]+k_{\text{DAD}}[\text{DA}]-(k_{\text{DDA}}+k_{\text{DF}})[D]$$ (2)

$$\frac{d[\text{FA}]}{dt}=k_{\text{FFA}}{\left[F\right]}^{2}-(k_{\text{FAF}}+k_{\text{FADA}}\cdot \xi )[\text{FA}]$$ (3)

$$\frac{d[\text{DA}]}{dt}=-k_{\text{DAD}}[\text{DA}]+k_{\text{DDA}}[D]+k_{\text{FADA}}\cdot \xi [\text{FA}]$$ (4)

$$\xi =1-\frac{\left[D]+[\text{DA}\right]}{\left[T\right]}$$ (5)

In these equations, [F], [FA], [D], and [DA] represent the number densities of assemblers in each of the four states. [*T*] and ξ are the number density of template positions and the fraction of unoccupied template positions, respectively. The rate constant of each transition is denoted as *k_xy_*, with *x* as the initial state and *y* as the final state, e.g., *k*_D − DA_ describes the rate of template-guided lateral association, while *k*_DA − D_ governs the reverse process of dissociation along the template. For the sake of simplicity, we have chosen to neglect several transitions, which are not likely to occur. The cross-transitions FA → D and DA → F involve the simultaneous detachment or attachment of multiple assemblers from the template coupled with simultaneous (de-)aggregation. As this requires many supramolecular reactions to occur within a short time period, it is not likely to contribute significantly to the process. In addition, the transition DA → FA is negligible in most cases as the multivalency of an assembled state gives rise to a strong cooperativity suppression of simultaneous detachment. We describe the underlying assumptions and their validity in more detail in the Supplementary Materials.

Within this complex reaction network, two main pathways lead the system toward the final docked-assembled (DA) state: (Pathway I: F → D → DA) Assemblers first bind the template, after which they laterally assemble, the pathway of true templated assembly, or (Pathway II: F → FA → DA) by first forming aggregates in solution, after which the aggregate recruits a template with its exposed docking domains. While the first pathway is the route envisioned in designing a templated assembly system, pathway II is often relevant and can lead to aggregates that are kinetically trapped and can steer the system to a nonequilibrium and dysfunctional state. It is our aim to understand how the design of the system and its interactions can be used to gain pathway selectivity and, ideally, to completely suppress any pathway, such as F → FA → DA, that can lead to kinetic trapping.

As the reaction network sketched above is governed by a plethora of unknown rate constants, we use a molecular model based on Brownian dynamics simulations to understand how the reaction network responds to changes in the molecular properties of the system. The simulation procedure is described in detail in Materials and Methods. We model the assembler and template as flexible polymers according to the Kremer-Grest bead-spring model (*23*). Our assembler species consists of three types of monomers, designed to match the corresponding stability (S), assembly (A), and docking (D) domains. The template is modeled as a longer Kremer-Grest polymer consisting of 90 template (T) beads. The attractive interactions between the assembly domains, interactions AA, are described by an attractive Lennard-Jones pair potential *U*_AA_, in which the strength of the interactions is set by the energy scale *E*_AA_. The binding interactions between the docking domains and the template, interaction DT, are modeled with an inverse Gaussian pair potential *U*_DT_, controlled by the energy scale *E*_DT_. This choice ensures that each template position can only bind a single docking monomer such that the stoichiometry of the binding is well controlled. The stoichiometry of assembler and template binding sites is a crucial aspect of virtually all templated assembly systems, e.g., the RNA template encapsulated by the tobacco mosaic virus has a limited number of negative charges that can be compensated by positively charged capsid proteins, leading to an exact preferred binding stoichiometry. While minimal, our model captures this important feature. All other interactions are described by short-ranged repulsive Weeks-Chandler-Andersen (WCA) potentials. The stability domains S only have repulsive interactions, both with themselves and all other domains, and thus act as steric groups that balance the associative interactions in the system.

### Pathway selection by balancing supramolecular interactions

Of the two primary pathways from free molecules in solution to a self-assembled nanostructure, pathway I is preferred. This path makes full use of the template as a coordinating species of assembly and, unlike pathway II, is less likely to result in kinetically trapped states, which can severely delay the assembly process and result in structural polymorphism. A mixture of both pathways would lead to a polymorphous end product. The central question is thus: How can we tune the design of the system to steer the assembly process toward pathway I, F → D → DA?

Our choice for the interaction potentials conveniently allows us to regulate the assembler-assembler and assembler-template with two control dials: *E*_AA_ and *E*_DT_, respectively. To elucidate their effects on the kinetic pathways, we perform Brownian dynamics simulations in which we vary *E*_AA_ and *E*_DT_ independently. As there are multiple supramolecular forces at play, templated assembly systems undergo a large number of elementary supramolecular reaction steps. As a result, the assembly process is characterized by many transient intermediate states (*24*).

**Fig. 2:**
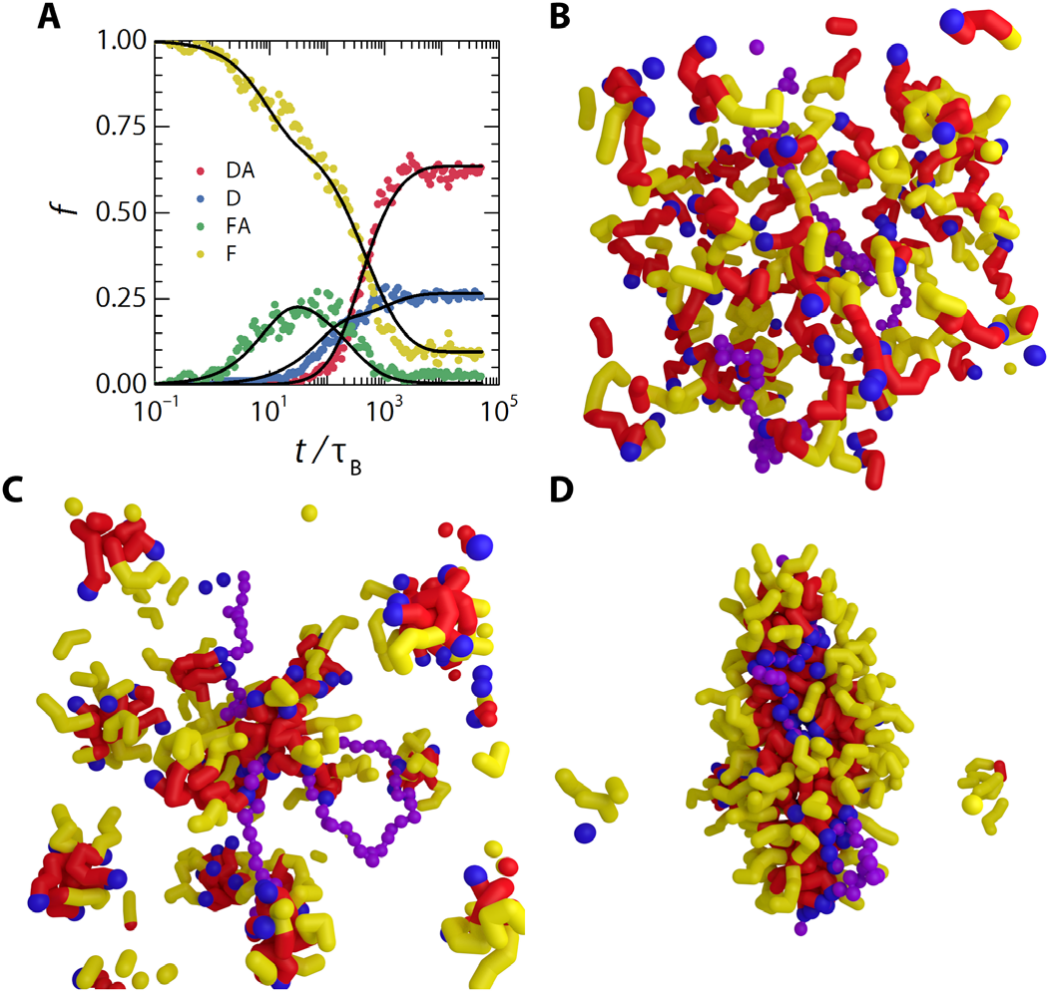
Monitoring templated self-assembly over time. (**A**) Typical kinetic diagram showing the evolution of the fraction of assemblers, *f*, in each of the four states over time. Simulation performed at *E*_AA_ = 1.0 *k*_B_*T* and *E*_DT_ = 17 *k*_B_*T*. (**B** to **D**) The general picture of simulations of the assembly process starts with a homogeneous field of assembler units and a single template (purple beads) (B); after some time, there appear free-assembled assembler species in solution, and there are clumps of assemblers on the template—the changes in template morphology as a direct result of assembled assemblers is visible (C). At the end of the simulation, most assemblers will have attached to the template and fully cover a highly deformed template (D). All simulations are performed in a box with periodic boundary conditions.

To classify these, we assign to each assembler in our system one of the four states illustrated in Fig. 1. For selected time points in the simulation, we determine what fraction *f* of the assemblers is present in each of these different states; for details on the categorization, we refer to Materials and Methods. The formation of the final DA state emerges in time as a sigmoidal curve in the resulting kinetic diagrams (Fig. 2A). Such a sigmoidal growth of the nanostructures toward their final architecture is typical for templated assembly and observed previously in both simulations and experiments (*25*–*27*). This is usually interpreted by the existence of three distinct phases. First, there is a slow initiation phase, during which intermediate structures are formed, and which is governed by diffusion-limited kinetics. Second, the assembly accelerates due to the cooperativity of the process. Third, the system approaches a steady state as the concentrations of free assemblers in solution decreases. In our results, the initiation coincides with a small number of assemblers that bind individually onto the template, forming a docked (D) fraction (Fig. 2A). Before the formation of the DA structure, we observe the formation of an aggregate fraction in the FA state, which later decreases as the aggregates dock and transition into a DA structure. In addition, cooperativity in the template binding, mediated by the lateral interactions of the assembly domains (A), emerges in our simulations through the formation of binding nuclei that grow in time to encapsulate the template (see the Supplementary Materials). The initial lag time in the sigmoidal kinetic curve could well be related to detachment kinetics of individual assemblers to the template. The formation of a packed assembled structure on the template requires a stable nucleus of docked assemblers to be formed first. If the detachment rate of individually docked assemblers is high, one can expect a long initial lag time before such a nucleus is formed. These initial lag times due to reversible attachment and detachment kinetics have been previously observed in colloidal self-assembly with weak attractive interactions (*28*).

**Fig. 3:**
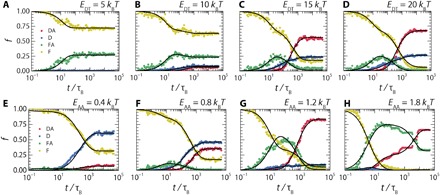
Tuning interaction strength steers the assembly kinetics. Kinetic diagrams are shown for a series of simulations with varying assembler-template attraction strengths (*E*_DT_) (**A** to **D**) and varying assembly-assembly (lateral associative) attraction (*E*_AA_) (**E** to **H**). We show the evolution of the fraction of assemblers *f* in each of the four assembly states over time *t*/τ_B_. Black lines represent the optimal solutions found for the kinetic model. Plotted for *E*_DT_ = 5, 10, 15, and 20 *k*_B_*T* (A to D) and *E*_AA_ = 0.4, 0.8, 1.2, and 1.8 *k*_B_*T* (E to H).

In our simulations, we increase the binding energy of the docking domain to the template, *E*_DT_, from 5 to 21 *k*_B_*T*, at a constant assembly-assembly domain attraction strength of *E*_AA_ = 1.0 *k*_B_*T*. Kinetic diagrams for four representative values of *E*_DT_ (others shown in the Supplementary Materials) are shown in Fig. 3 (A to D). As we increase *E*_DT_, we observe an increase in the fractions of assemblers in the docked and DA states. At higher values of *E*_DT_, we observe a depletion of the free-assembled (FA) fraction at later time scales as the aggregates start docking onto the template. The maximum observed fraction of assemblers in the FA states remains constant at 0.3, indicating that the formation of freely assembled structures in solution remains unaffected by *E*_DT_. This is expected, as this is driven solely by the lateral interactions set by *E*_AA_. At weak template binding strengths, the aggregated template-free state in solution is the stable final product, while stronger interactions with the template lead to template recruitment of these aggregates and transform them into DA state through undesired pathway II, F → FA → DA.

We can quantify the influence of template binding strength on the templated assembly process, for all values of *E*_DT_, by fitting our kinetic diagrams to our analytical reaction equation model. We solve the kinetic reaction model using the Runge-Kutta method and fit it to the simulation data using a simulated annealing algorithm, as described in more detail in Materials and Methods. We find that the simplified reaction model describes the simulation data with rather high precision (see lines in Fig. 3). Thus, while the reaction model in Eq. 1 is approximate, it appears to capture the governing states and pathways. From the quantitative mapping of the model onto the simulation results, we gain access to the rate constants that govern the primary supramolecular reaction steps.

**Fig. 4:**
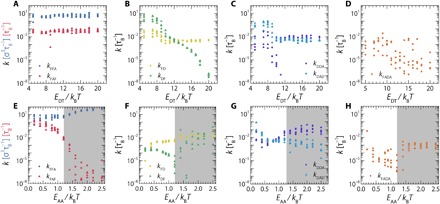
Evolution of rate constants over varying interaction strengths. Rate constants found for the kinetic model are shown over a series of simulations with increasing assembler-template interaction strength (*E*_DT_) (**A** to **D**) and increasing assembly-assembly attraction strength (*E*_AA_) (**E** to **H**). Data points are the average value of the 10 best fits found by the simulated annealing optimization algorithm. We performed each simulation five times and have plotted results for all replicates as an indicator of the spread between identical simulations. The gray shaded area in (E) to (H) represents the values for *E*_AA_ ( ≥ 1.2 *k*_B_*T*), where we find substantial aggregates in bulk and our model starts to loose accuracy.

We find that the rate constants *k*_FFA_ (F → FA) and *k*_FAF_ (FA → F) remain within the same order of magnitude (Fig. 4A), confirming our observation that the formation of FA structures in solution is unaffected by the assembler-template interactions. As *E*_DT_ increases from 5 to 20*k*_B_*T*, the undocking rate *k*_DF_ (D → F) decreases by more than six orders of magnitude (Fig. 4B). Between *E*_DT_ 8 and 18 *k*_B_*T*, this rate constant appears to decrease exponentially, which is in agreement with Arrhenius behavior expected for a thermally activated process. By contrast, the forward docking rate *k*_FD_ is virtually constant as a function of *E*_DT_, as the F → D transition is rate-limited by diffusion. Notably, for rate constants *k*_FD_, *k*_DF_, *k*_DDA_, and *k*_DAD_, we find high standard deviations and fluctuating rate constants for *E*_DT_ between 5 and 10 *k*_B_*T* (Fig. 4, B and C). In this regime, the fractions of assemblers in the docked and DA states are very low (see Fig. 3) such that the fit gives results with a very low confidence. As *E*_DT_ > 10 *k*_B_*T*, both *k*_DDA_ and *k*_DAD_ become insensitive to *E*_DT_, as expected. Last, we note that the rate constant *k*_FADA_ (FA → DA), describing the transition where large aggregates in solution bind a template molecule in one go, which is the template recruitment of aggregates as mentioned before, shows strong scatter. This is due to the simplification in our model that this reaction step is controlled by a single rate constant; in reality, this process is diffusion-limited and thus depends on the size of the aggregates, which will show time evolution and distribution. While for the sake of simplicity, we chose here not to incorporate this complexity in our model, this could, in principle, be done by introducing a diffusion-limited aggregation kernel into the kinetic rate equations (*29*).

Upon increasing *E*_DT_, the equilibrium of the reversible reaction F → D is shifted toward the right, while that of F → FA and D → DA is largely unaffected. As a result, the fraction of assemblers that freely docks onto the template is accelerated, while the number of assemblers intermediately trapped in the FA state remains constant. From these observations, we can extract a first design rule: Increasing the assembler-template attraction can be used to accelerate the favorable assembly pathway I, with respect to pathway II that occurs through solution aggregation. Thus, while this is a promising route toward pathway selectivity, we find that, given the parameter space explored here, a complete suppression of pathway II is not possible by tuning the template binding strength alone.

Because the FA state that we would like to suppress is mediated by the AA interaction, we turn our attention to pathway selection by tuning *E*_AA_. We perform a series of simulations with increasing assembler-assembler interaction strengths from *E*_AA_ = 0.2 *k*_B_*T* to *E*_AA_ = 2.4 *k*_B_*T* at strong assembler-template interactions, *E*_DT_ = 17 *k*_B_*T* (Fig. 3, E to H); full kinetic diagram can again be found in the Supplementary Materials. In addition, we compare the results from Brownian dynamics simulations with our kinetic reaction network model to evaluate how pathway selectivity can be attained. Between *E*_AA_ = 0.2 *k*_B_*T* and *E*_AA_ = 1.0 *k*_B_*T*, which correspond to overall lateral pair interactions strengths of 1 to 5 *k*_B_*T* as our model features five monomers in each A domain, our simulation results are well described by our model. Over the course of this series, we observe increasing fractions of assemblers in the FA state, corresponding to the formation of more assembler aggregates. This trend is visible in the rate constants we obtain from our kinetic model, where the rate constant *k*_FAF_, which describes the dissociation of assemblers from the FA state, decreases by more than an order of magnitude (Fig. 4E). At these moderate assembly-assembly attraction strengths, we observe an increase in the amount of assemblers tightly packed on the template (DA) and a corresponding reduction in the amount loosely packed on the template (D). This trend highlights the necessity of lateral interactions between assemblers for tight packing of assemblers on the template and indicates that, while *E*_AA_ steers the assembly process mainly via pathway II, it also contributes positively to the second stage of pathway I, where docked units assemble further on the template via lateral attractions.

Stronger assembler-assembler attraction also causes adverse effects, as it promotes the formation of aggregates in the FA state that only slowly dock onto the template, as this process is diffusion-limited and is thus inversely proportional to the aggregation number of the solution clusters (Fig. 3G). The aggregates become more pronounced as we increase the assembly-assembly attraction to *E*_AA_ values upward of 1.0 *k*_B_*T*. Under these conditions, our kinetic diagrams show a much slower assembly process. We observe large transient fractions of assemblers in the FA state that transition toward the DA state in a stepwise fashion (Fig. 3H), where every step corresponds to the docking event of a cluster. The formation of these large aggregates can also be observed in the simulation itself (movie S1).

At the point where aggregates start to dominate the kinetics, *E*_AA_ > 1.2 *k*_B_*T*, the assembly process is no longer captured by our model, as the assumption that the FA → DA transition is a first-order process that relies on the single rate constant *k*_FADA_ no longer holds: Our simulations show a range of aggregate species with varying sizes and hence varying mobilities.

From these results, we conclude that increasing *E*_AA_ has a two-faced effect on the assembly process: On the one hand, it leads to a denser capsid around the template, as templated assembly through pathway I is promoted by strong lateral interactions of docked assemblers, while, on the other hand, promoting the formation of solution aggregates that slow down the assembly process and promote the relative occurrence of undesirable pathway II. This leads us to a second design rule: While lateral interactions are essential to create templated assembly and a dense cohesive structure, it must be moderated to avoid excessive aggregation in solution that could lead to kinetic trapping and polymorphism.

However, strong interaction energies do not necessarily lead to trapped intermediate states, depending on the interaction being tailored. While strong assembler-assembler interactions lead to aggregation in solution and, consequently, the breakdown of the kinetic model, strong assembler-template interactions, in fact, push the system toward the preferred pathway (pathway I) without inducing nontemplated aggregates. This effect occurs even though detailed balance is broken, as the assembler undocking rate (*k*_DF_) is multiple orders of magnitude lower than the assembler docking rate (*k*_FD_) at high *E*_DT_ (Fig. 4B).

We note, in passing, that other factors besides these interaction energies might play a role in tuning the pathways of templated assembly. One example is template flexibility. Besides the simulations discussed here, which contain a flexible template, we have also run simulations in which the template is entirely rigid, as shown in the Supplementary Materials. If template folding is entirely suppressed, this inevitably leads to nanostructure geometries that are dictated by the template, rather than by the assemblers. This implies that tuning template flexibility between the two extremes of entirely flexible and rigid might result in a range of potential structures. Our model system could be readily adapted to include a bond-bending potential to mimic semiflexible polymers, which makes the effect of template flexibility an interesting avenue for future study.

### Allosteric control of templating pathways

From the results discussed in the previous section, it is clear that, while some selectivity in pathways can be obtained by tailoring the balance of supramolecular interactions in the system, complete suppression of the undesired kinetic pathway II is not possible. The two-sided nature of the assembler-assembler interaction strength means that the range of *E*_AA_ energies that can be used when designing effective templating systems is restricted. Interaction strengths should promote lateral interactions and thus promote dense packings on the template but should also aim to minimize the aggregation of assemblers in bulk. Nature has developed clever design strategies, e.g., in templated assembly of many RNA viral capsids, to circumvent these limitations (*30*, *31*). One such mechanism is allostery, where capsid proteins switch conformation as they bind to the RNA template that activates their lateral interactions (*32*, *33*). Thus, the assembler-assembler interactions only become active upon binding their template. This strategy allows high attraction strengths between the assemblers to ensure a dense and cohesive nanostructure, without the side effect of aggregation in solution. Despite the widespread use of allostery in natural systems, it remains to be harnessed to gain pathway selectivity in synthetic templated assembly systems. To explore how allosteric control could be used to select the kinetic pathway of interest, we introduce a coarse-grained allosteric mechanism into our simulations.

**Fig. 5:**
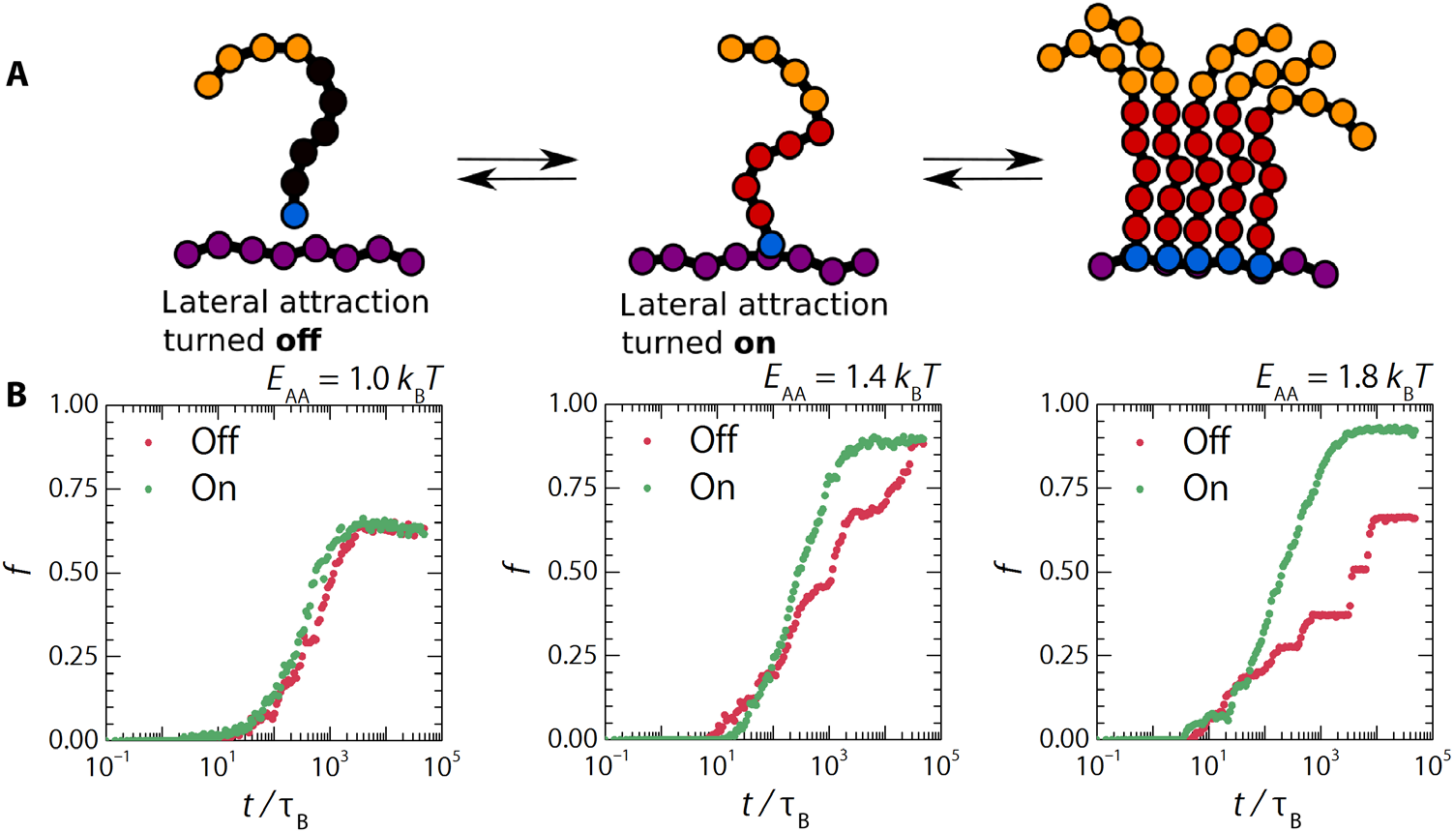
Allosteric switching accelerates templated self-assembly. (**A**) Mechanism of allostery defined in the simulations. (**B**) Kinetic diagrams showing the fraction of assemblers (*f*) in the docked-assembled (DA) state over time, with allostery both turned on (green) and off (red) for the three values of *E*_AA_. Simulations were carried out at *E*_DT_ = 17 *k*_B_*T*.

We model allostery as a binary switch that toggles the attractive interactions between the assembly domains from off to on, depending on whether assemblers are docked to the template (Fig. 5A). During the simulation, we track for each assembler if it is in solution (F and FA states) or docked to the template (D and DA states). If an assembler is in solution and the allosteric mechanism is not activated, the assembly domains are repulsive by means of a WCA potential. Upon docking, the allosteric switch changes the identity of these monomers in the assembler from repulsive to attractive.

To evaluate the effectiveness of allostery as a selection mechanism for the desired assembly pathway, we compare the formation rate of the DA fraction in the presence and absence of allostery at varying assembler-assembler attraction strengths (Fig. 5B). At weak assembler-assembler attractions (*E*_AA_ = 1.0 *k*_B_*T*), we observe little difference between simulations with and without allostery. However, at greater *E*_AA_ of 1.4 and 1.8 *k*_B_*T*, allostery greatly reduces the time required for assembly by suppressing the formation of slow and potentially kinetically trapped solution aggregates. We no longer find the stepwise assembly that occurs in the absence of allostery, through the template recruitment of large aggregates, but rather observe a smooth assembly process that occurs exclusively through the desired pathway I.

**Fig. 6:**
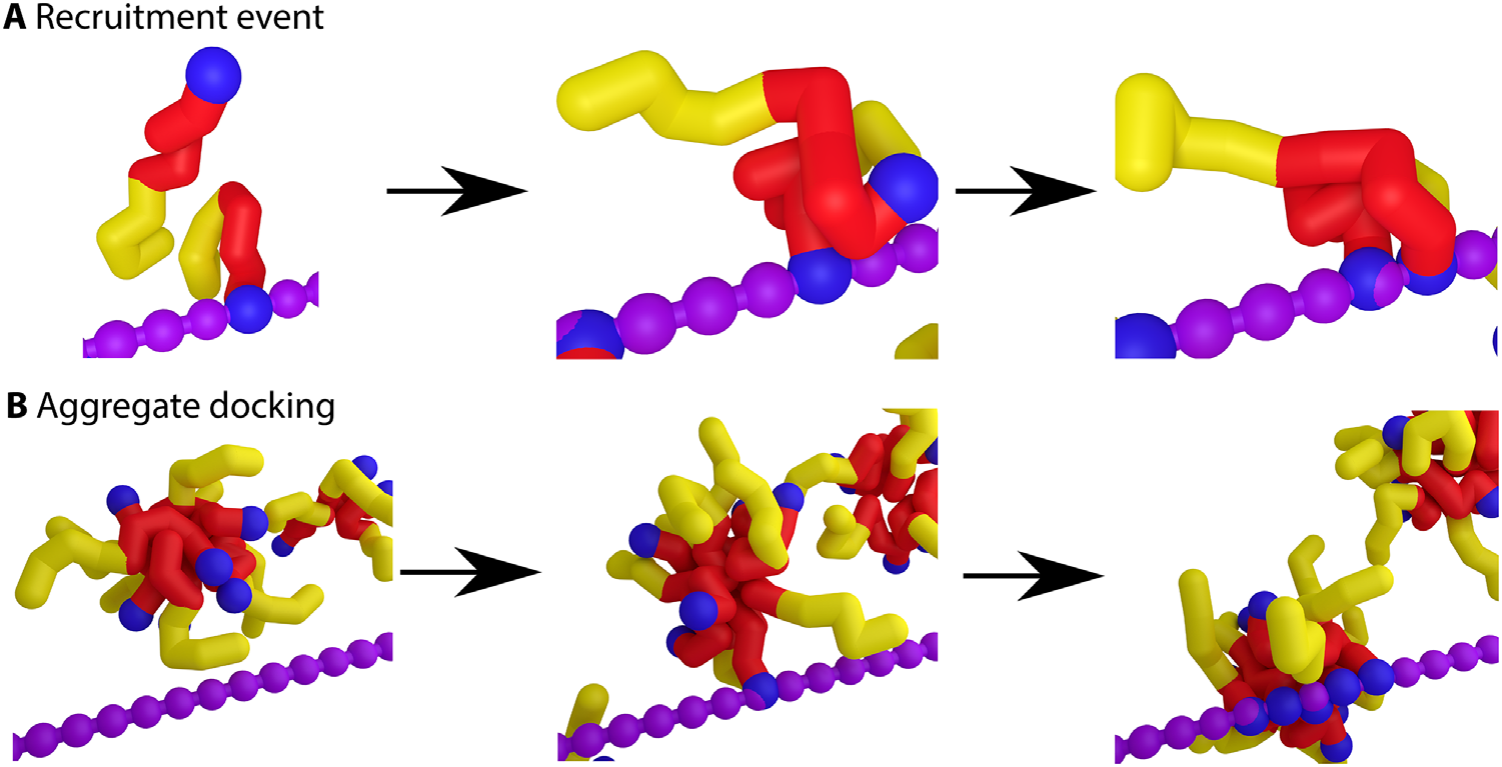
Two observed assembly mechanisms. (**A**) Simulation snapshot of a recruitment event of an individual assembler by one already docked on to the template. (**B**) Snapshot of a collective docking event of an aggregate of assemblers. These simulations have been performed on a static and stretched template as an aid to visual clarity. All other data in this paper use a fully flexible template as described in Materials and Methods.

To understand how this works, we visualize the assembly from simulation snapshots. In the absence of allostery, we observe many aggregate docking events for strong assembler-assembler attractions (Fig. 6A). These events characteristically show the initial docking of a single assembler within the aggregate, followed by a structural reorganization that results in docking of the remainder of the assemblers. If allostery is introduced, the formation of these aggregates is completely suppressed. This effect is visible as we compare simulations in the absence and presence of allostery (movies S1 and S2, respectively). Instead of aggregate docking, we observe many assembler-recruitment events, where assemblers bind to assemblers already docked onto the template and subsequently reorganize to dock onto the template themselves (Fig. 6B). These recruitment events lead to a transient semi–FA state, as can be observed in the kinetic diagrams of the simulations performed with allostery (fig. S5). We performed a cluster analysis to differentiate between those assemblers in the true FA state and those that are indirectly attached to the template (section S4). Using this cluster analysis, we find that, for our simulations with allostery, all assemblers in the FA state are indirectly attached to the template, and no freely diffusing aggregates are present (fig. S8). In contrast, in our simulations without allostery, we do identify large fractions of assemblers that are part of aggregates (fig. S8). Allostery, as used by nature, is thus an exquisite strategy for pathway selectivity in templated assembly.

## DISCUSSION

In this work, we presented a minimal simulation model to capture the essential and generic features of templated self-assembly. Being a simplified model, we have chosen to work with relatively unspecific potentials. As such, one may expect the outcomes to be generic in nature. Although using more specific potentials with different shapes would affect the precise values of the rate constants in our model, the overall picture remains, allowing us to extract several important and novel insights. Our results shed new light on the pathways involved in templated assembly and how these pathways can be controlled through chemical design. We have revealed how tuning the balance between the interactions between the multivalent template and the building blocks that it binds can be used to gain some level of control over the pathways in which the system evolves. However, the lateral interactions between building blocks, which are a necessity to create a cooperative assembly process that leads to a dense and cohesive nanostructure, also invariably lead to the formation of solution aggregates that slow down the process, introducing kinetically trapped states and structural polymorphism. We have shown how nature’s use of allosteric activation is ideally suited to suppress all undesired pathways and lead to excellent pathway selectivity in the assembly process. To date, no synthetic realization for allosteric control of templated assembly exists, and several building blocks to realize synthetic allostery have been developed (*34*, *35*), which may be used to go toward truly biomimetic templating strategies to create nanostructures with high fidelity using our design model as a rational basis. Moreover, the design model we have introduced here for a flexible one-dimensional template could be readily adapted to explore how other design factors in templated assembly, such as the template flexibility and dimensionality, assembler geometry, stoichiometry, and overall reactant concentrations, could steer the kinetic pathways and resulting end products. This would make for a rational design basis to create synthetic systems in which desired nanostructures can be formed through templated assembly with high fidelity.

## MATERIALS AND METHODS

### Simulations

We performed Brownian dynamics simulations using the HOOMD-blue v2.2.0 package (*36*, *37*). Quantities are expressed in reduced units, in terms of the monomer diameter σ, the monomer self-diffusion time τ_B_, and *k*_B_*T* as the characteristic energy scale. All simulations contain two molecular species: The first is a single template consisting of 90 template domains (*T*_90_), and the second species are 96 assemblers containing solubility domains (S), assembly domains (A), and a docking domain (D) in conformation *S*_4_*A*_5_*D*_1_. All connecting bonds were defined according to the Kremer-Grest bead-spring model, in which neighboring particles are connected by a FENE spring, defined as (*23*)

$$U_{\text{FENE}}(r)=-\frac{1}{2}kr_{0}^{2}\text{ln}(1-{\left(\frac{r}{r_{0}}\right)}^{2})+V_{\text{WCA}}(r)$$ (6)

where *k* = 30 *k*_B_*T*/σ^2^ is the attractive force strength, *r* denotes the center-to-center distance between two bonded domains, *r*_0_ = 1.5σ is the bond size parameter, and *V*_WCA_(*r*) is a repulsive WCA potential

$$U_{\text{WCA}}(r)=\{\begin{array}{ll} 4\varepsilon [{\left(\frac{\sigma }{r}\right)}^{12}-{\left(\frac{\sigma }{r}\right)}^{6}]+\varepsilon & \;r<2^{\frac{1}{6}}\sigma \\ 0 & \;r\geq 2^{\frac{1}{6}}\sigma \end{array}$$ (7)

Here, ε = 1 *k*_B_*T* is the characteristic energy and *r* = 2^1/6^ σ is the interparticle distance where the potential is zero.

For the assembled-assembler attraction, we used the Lennard-Jones pair potential

$$U_{\text{AA}}=4E_{\text{AA}}[{\left(\frac{\sigma }{r_{\text{AA}}}\right)}^{12}-{\left(\frac{\sigma }{r_{\text{AA}}}\right)}^{6}]$$ (8)

with −*E*_AA_ as the attraction strength of the Lennard-Jones potential and *r*_AA_ as the center-to-center distance between the assembly domains.

For the interaction between the docking domain and the template, we used an inverted Gaussian pair potential described by

$$U_{\text{DT}}=-E_{\text{DT}}\cdot S(r)\cdot \text{exp}[-\frac{1}{2}{\left(\frac{r_{\text{DT}}}{0.25\sigma }\right)}^{2}]$$ (9)

Here, *r*_DT_ is the docking template domain interparticle distance and −*E*_DT_ is the minimum of the potential at *r* = 0σ. *S*(*r*) is a smoothing function that ensures that the pair potential transitions smoothly to 0 at large values of *r*. *S*(*r*) operates from *r*_on_ = 0.4σ to *r*_cut_ = 0.5σ as follows

$$S(r)=\{\begin{array}{ll} 1 & r<r_{\text{on}}\\ \frac{{\left(r_{\text{cut}}^{2}-r^{2}\right)}^{2}\cdot (r_{\text{cut}}^{2}+2r^{2}-3r_{\text{on}}^{2})}{{\left(r_{\text{cut}}^{2}-r_{\text{on}}^{2}\right)}^{3}} & r_{\text{on}}\leq r\leq r_{\text{cut}}\\ 0 & r>r_{\text{cut}} \end{array}$$ (10)

The interactions between all remaining particle pairs are described by the repulsive WCA potential (Eq. 7).

Simulations were performed in the canonical ensemble by integrating the overdamped Langevin equation using time steps of 10^−4^ τ_B_ (*38*). In our simulations with a flexible template, assemblers were initially positioned on a cubic lattice within the simulation box of dimensions 25 × 25 × 25 σ, with the template localized in a spiral conformation in the center. Before the measurement, the initial conformations were equilibrated for a period of 10^3^ τ_B_, where both the A-A Lennard-Jones and the D-T inverse Gaussian potentials were replaced by a WCA potential, to suppress attractive interactions and allow the system to reach a randomized configuration. Consecutively, simulations were carried out for a period of 5 × 10^4^ τ_B_. Simulation snapshots were stored every 0.05 τ_B_ for the initial 10 τ_B_ of simulation time and 0.5 τ_B_ for the remainder to capture processes that occur on both short and long time scales with a sufficiently high time resolution.

We incorporated allostery in our simulations with the use of a callback function. Every 0.1τ_B_, the positions of the docking domains and template positions were compared. If the docking domain of an assembler is not within a distance of 0.5σ from a template position, its assembly domains are turned inactive, replacing the attractive Lennard-Jones pair potential governed by *E*_AA_ by a purely repulsive, hard sphere–like, WCA pair potential. If the docking domain of an assembler is within this target distance of a template position, this process is reversed. Simulations with allostery were performed on a flexible template, with *E*_DT_ = 17 *k*_B_*T* (Fig. 6B).

### Analysis

We determined the fraction of assemblers in each of the four states (free, free assembled, docked, and docked assembled) over time by assessing for each snapshot whether each assembler was docked to the template, attached to other assemblers, or both. Assemblers were counted as docked when they were within 0.5σ distance from a template domain. Similarly, they were counted as assembled when at least four pairs of A domains with a neighboring assembler were within a distance of 1.3σ. The criterion of four domains was chosen to distinguish between assemblers that were firmly assembled and those that were coincidentally in each other’s proximity but not firmly bonded. The data were temporally averaged in 2000 logarithmic spaced bins along the entire length of the simulation.

### Solving the kinetic model and fit algorithm

We solved the kinetic model numerically using the Runge-Kutta method, with step size d*t* = 0.01 τ_B_ for the initial 10 τ_B_ of the simulation and d*t* = 0.25 τ_B_ for the remainder. Parameters for the kinetic model were found by minimizing the sum of squared residuals (*R*) between the kinetic diagrams and the kinetic model for each binned data point

$$R=\sum_{i=1}^{n}[{\left({\left[F\right]}_{i}-f_{F,i}\right)}^{2}+{\left({\left[\text{FA}\right]}_{i}-f_{\text{FA},i}\right)}^{2}+{\left({\left[D\right]}_{i}-f_{D,i}\right)}^{2}+{\left({\left[\text{DA}\right]}_{i}-f_{\text{DA},i}\right)}^{2}]$$ (11)

where *n* = 2000 equals the number of logarithmic spaced binned data points, [FA]*_i_* is the number density of assemblers in the FA state at data point *i*, and *f*_FA, *i*_ is the concentration as obtained from the kinetic model at this data point. *R* was minimized using a simulated annealing fit algorithm with an exponential multiplicative cooling schedule as described by Kirkpatrick and coworkers (*39*). For every run of the algorithm, we performed 500 cooling steps, starting from an initial effective temperature *T*_0_ = 10, with a cooling factor of α = 0.97. At every cooling step, the seven fit parameters (*k*_FFA_, *k*_FAF_, *k*_FD_, *k*_DF_, *k*_DDA_, *k*_DAD_, and *k*_FADA_) were changed randomly using the NumPy random number generator. As the simulated annealing algorithm proceeds to lower temperatures, the tolerance for unfavorable steps decreases, lowering the acceptance rate. To ensure that sufficient meaningful parameter adjustments were made, we continuously changed the parameter adjustment step size to keep the acceptance rate at 0.5. Our use of seven fit parameters resulted in a complex goodness-of-fit landscape with many possible local minima. To find the global minimum in this landscape, we repeated the simulated annealing algorithm 100 times starting from randomized initial parameter values. Average parameter values and standard deviations were computed for the best 10 fits obtained in this way. Corresponding residuals are presented in section S5.

## Supplementary Material

http://advances.sciencemag.org/cgi/content/full/5/10/eaaw3353/DC1

Download PDF

Movie S1

Movie S2

Allosteric pathway selection in templated asembly

## SUPPLEMENTARY MATERIALS

Supplementary material for this article is available at http://advances.sciencemag.org/cgi/content/full/5/10/eaaw3353/DC1 Section S1. Assumptions made in the kinetic model Section S2. Cooperativity without allostery Section S3. Overview of all kinetic diagrams Section S4. Differentiating between aggregates and recruited assemblers Section S5. Residuals obtained for the simulated annealing fit algorithm Fig. S1. Determining the reaction order of free assembly. Fig. S2. Template occupancy profiles for simulations with a rigid template. Fig. S3. Overview of kinetic diagrams at varying assembler-template interaction strengths without allostery. Fig. S4. Overview of kinetic diagrams at varying assembler-assembler interaction strengths without allostery. Fig. S5. Overview of kinetic diagrams at varying assembler-assembler interaction strengths including allostery. Fig. S6. Schematic representation of two free-assembled (FA) states. Fig. S7. Overview of kinetic diagrams at varying assembler-assembler interaction strengths without allostery, differentiating between two FA states. Fig. S8. Overview of kinetic diagrams at varying assembler-assembler interaction strengths including allostery, differentiating between two FA states. Fig. S9. Histograms of fit residuals at varying assembler-template interaction strength. Fig. S10. Histograms of fit residuals at varying assembler-assembler interaction strength. Movie S1. Time lapse of a simulation in the absence of allostery. Movie S2. Time lapse of a simulation including allostery. References (*40*–*46*)
