# Supplementary material for: Allosteric pathway selection in templated assembly
Source: Sci Adv. 2019 Oct 11;5(10):eaaw3353. doi: 10.1126/sciadv.aaw3353 (PMC6788871; doi:10.1126/sciadv.aaw3353)
Supplement: http://advances.sciencemag.org/cgi/content/full/5/10/eaaw3353/DC1 [file supp_5_10_eaaw3353__index.html]

Science Advances | Science AdvancesAAASSearchScience AdvancesMenu

## Supplementary Materials

**The PDF file includes:**

- Section S1. Assumptions made in the kinetic model
- Section S2. Cooperativity without allostery
- Section S3. Overview of all kinetic diagrams
- Section S4. Differentiating between aggregates and recruited assemblers
- Section S5. Residuals obtained for the simulated annealing fit algorithm
- Fig. S1. Determining the reaction order of free assembly.
- Fig. S2. Template occupancy profiles for simulations with a rigid template.
- Fig. S3. Overview of kinetic diagrams at varying assembler-template interaction strengths without allostery.
- Fig. S4. Overview of kinetic diagrams at varying assembler-assembler interaction strengths without allostery.
- Fig. S5. Overview of kinetic diagrams at varying assembler-assembler interaction strengths including allostery.
- Fig. S6. Schematic representation of two free-assembled (FA) states.
- Fig. S7. Overview of kinetic diagrams at varying assembler-assembler interaction strengths without allostery, differentiating between two FA states.
- Fig. S8. Overview of kinetic diagrams at varying assembler-assembler interaction strengths including allostery, differentiating between two FA states.
- Fig. S9. Histograms of fit residuals at varying assembler-template interaction strength.
- Fig. S10. Histograms of fit residuals at varying assembler-assembler interaction strength.
- References (*40*–*46*)

Download PDF

**Other Supplementary Material for this manuscript includes the following:**

- Movie S1 (.mp4 format). Time lapse of a simulation in the absence of allostery.
- Movie S2 (.mp4 format). Time lapse of a simulation including allostery.

**Files in this Data Supplement:**

- Adobe PDF - aaw3353\_SM.pdf
