# Supplementary material for: Allosteric pathway selection in templated assembly
Source: Sci Adv. 2019 Oct 11;5(10):eaaw3353. doi: 10.1126/sciadv.aaw3353 (PMC6788871; doi:10.1126/sciadv.aaw3353)
Supplement: Download PDF [file aaw3353_SM.pdf]

Science Advances
AAAS

advances.sciencemag.org/cgi/content/full/5/10/eaaw3353/DC1

Supplementary Materials for

Allosteric pathway selection in templated assembly

Martijn van Galen, Ruben Higler, Joris Sprakel*

*Corresponding author. Email: joris.sprakel@wur.nl

Published 9 October 2019, *Sci. Adv.* **5**, eaaw3353 (2018)
DOI: 10.1126/sciadv.aaw3353

The PDF file includes:

Section S1. Assumptions made in the kinetic model
Section S2. Cooperativity without allostery
Section S3. Overview of all kinetic diagrams
Section S4. Differentiating between aggregates and recruited assemblers
Section S5. Residuals obtained for the simulated annealing fit algorithm
Fig. S1. Determining the reaction order of free assembly.
Fig. S2. Template occupancy profiles for simulations with a rigid template.
Fig. S3. Overview of kinetic diagrams at varying assembler-template interaction strengths without allostery.
Fig. S4. Overview of kinetic diagrams at varying assembler-assembler interaction strengths without allostery.
Fig. S5. Overview of kinetic diagrams at varying assembler-assembler interaction strengths including allostery.
Fig. S6. Schematic representation of two free-assembled (FA) states.
Fig. S7. Overview of kinetic diagrams at varying assembler-assembler interaction strengths without allostery, differentiating between two FA states.
Fig. S8. Overview of kinetic diagrams at varying assembler-assembler interaction strengths including allostery, differentiating between two FA states.
Fig. S9. Histograms of fit residuals at varying assembler-template interaction strength.
Fig. S10. Histograms of fit residuals at varying assembler-assembler interaction strength.
References (*40*–*46*)

Other Supplementary Material for this manuscript includes the following:

(available at advances.sciencemag.org/cgi/content/full/5/10/eaaw3353/DC1)

Movie S1 (.mp4 format). Time lapse of a simulation in the absence of allostery.
Movie S2 (.mp4 format). Time lapse of a simulation including allostery.

Section S1. Assumptions made in the kinetic model

First, we neglect the cross-transitions $FA \rightarrow D$ and the $DA \rightarrow F$, since we aim to describe the assembly process with only the two pathways described. If they do occur, these cross transitions are expected to contribute little to the overall assembly process, since they involve simultaneous (un)docking and (dis)assembly events. For instance, the $F \rightarrow DA$ transition means an assembler simultaneously docks onto the template and attaches to other assemblers. While this could occur, we expect it is more likely that the these two events occur sequentially rather than simultaneously, meaning that the process as a whole takes place via either the FA or the D state.

Secondly, we presume the $DA \rightarrow FA$ transition is negligible. This process corresponds to the simultaneous release of a group of assemblers from the template, so that the energy barrier of this transition will be considerably greater than that of the other transitions in the model. We therefore assume the rate of this transition as negligible compared to the other rate constants in the network.

Thirdly, for the docking transitions $F \rightarrow D$ and $FA \rightarrow DA$, we assume that the assemblers are more mobile than the much longer template, so that these steps can be described as first-order reactions governed by the number density of assemblers. We further modulate these rates by the fraction of unoccupied template positions ξ. Each of the other transitions in our model is assumed to be a first-order process, with the exception of the $F \rightarrow FA$ transition.

Finally, we assume the transitions between the free and free assembled states ($F \rightarrow FA$) follow second-order kinetics. Supporting simulations with high E_{AA} in the absence of a template (Fig. S1) show that this process can be described with second order kinetics for the initial 30 τ_B, after which it gradually transitions into a first-order process. Initially, the $F \rightarrow FA$ process can be considered a bi-molecular reaction, governed by the diffusion rates of two assemblers. At later stages however, the transition is governed by diffusion of mobile individual assemblers to relatively immobile FA aggregates, which can be considered as a uni-molecular first-order process. As we find the $F \rightarrow FA$ process to be most prevalent in the initial stages of the simulation, we model the overall transition as a second order process. The following subsection in the Supplemental Information describes the simulations we performed to support this assumption.

Determination of the reaction order of free assembly

In our kinetic model, we make the assumption that the $F \rightarrow FA$ transition can be described by second order reaction kinetics. To test to what extent this assumption is valid, we perform supporting simulations designed such that all transitions besides the $F \rightarrow FA$ transition can be ignored. Our simulation box contains 1152 assemblers to ensure sufficient statistics. We do not include a template, so that we can ignore the $F \rightarrow D$ and $FA \rightarrow DA$ transitions. We further impose strong assembler-assembler attraction strengths at $E_{AA} = 10\ k_BT$ to ensure that any assemblers that have formed to freely-assembled aggregates will not be able to dissociate.

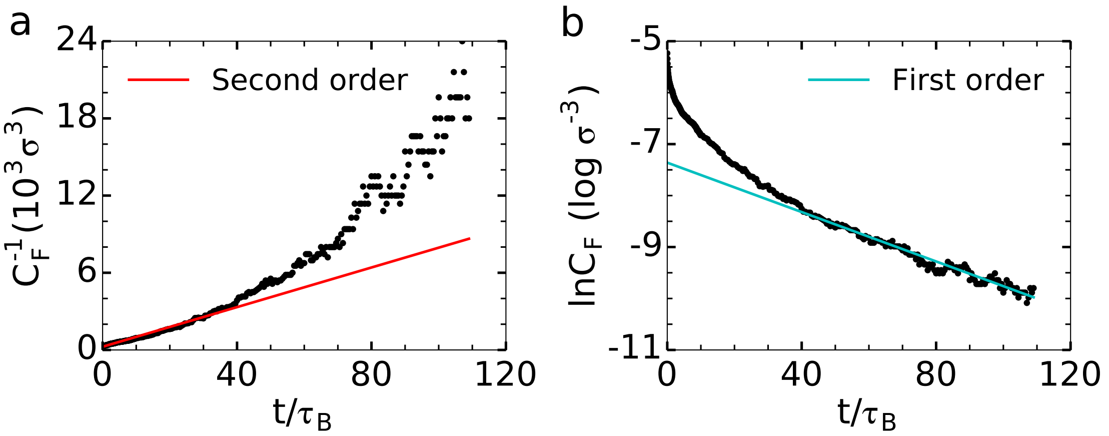

Fig. S1. Determining the reaction order of free assembly. Evolution of the concentration of assemblers in the F-state, C_F over time, as plotted for (a): second order kinetics (C_F^{-1}) and (b): first order kinetics ($ln\ C_F$), along with typical rate plots for second and first order kinetics.

This allows us to ignore the $FA \rightarrow F$ transition. All other simulation parameters were kept equal to those reported in the Materials & Methods section of the main paper. Effectively, this results in a state of diffusion-limited aggregation, in which we follow the decrease of the concentration of assemblers in the F-state (C_F). Plots of C_F^{-1} and $ln\ C_F$ over time are shown in figure S1, along with plots of second and first order rate equations. Over the course of the simulation, we observe a transition from second to first order kinetics, with a good agreement for second order kinetics in the initial 30 τ_B, and for first order kinetics after 40 τ_B. We can explain this transition as follows: In the initial stages the concentration of FA aggregates is low. As a result, the $F \rightarrow FA$ transition can be considered as a second order process, where two highly mobile assemblers (A) come together to form a single FA aggregate

$$A + A \longrightarrow A_2\ (2nd\ order\ in\ A)$$

At later stages large FA aggregates form. As a result the $F \rightarrow FA$ transition relies entirely on the diffusion of a single assemblers towards relatively immobile aggregates, which can be considered a first-order process

$$A + A_n \longrightarrow A_{n+1} \ (1st\ order\ in\ A)$$

Our kinetic diagrams show that the $F \rightarrow FA$ transition is most relevant in the initial stages of the simulations, since at those stages most of the FA-state is formed. We therefore consider this transition 2nd order in our kinetic model.

Section S2. Cooperativity without allostery

The simulations in this section are to show the emergence of cooperativity in the assembly process without allostery. To limit the effect of template flexibility, we keep the template in a fixed and linear conformation for these simulations. During these simulations we record whether each of the 90 template domains is occupied by the docking domain of an assembler. We express the results as the fractional occupancy, θ, of each position p over time.

Occupancy profiles for a series of measurements with varying E_{AA} at a high E_{DT} energy of 17 k_BT are shown in figure SS2, along with a snapshot of the final frame of each simulation. At low assembler-assembler interaction strength ($E_{AA} = 1.0\ k_BT$) we observe a random adsorption behaviour of assemblers on to the template, with little preference for docking adjacent to occupied template sites. Here the assembly behaviour tends towards Langmuir-like adsorption, which assumes that there are no interactions between adsorbed particles on adjacent sites (*41*). This type of adsorption behaviour breaks down as we increase the assembler-assembler strengths to $E_{AA} = 1.4\ k_BT$, and even more so at $E_{AA} = 1.8\ k_BT$. Rather than an assembly process that is evenly spread across the template, we observe docking via growing clusters, with a clear preference for assemblers to dock adjacent occupied template sites. Snapshots of the final simulation frame also reveal more condensed structures at $E_{AA} = 1.8\ k_BT$ when compared to $E_{AA} = 1.0\ k_BT$. Due to the strong assembler-assembler interactions, binding of assemblers in positions adjacent to docked assemblers becomes increasingly favourable, as compared to the initial binding of free assemblers. This behaviour is typical for positive binding cooperativity

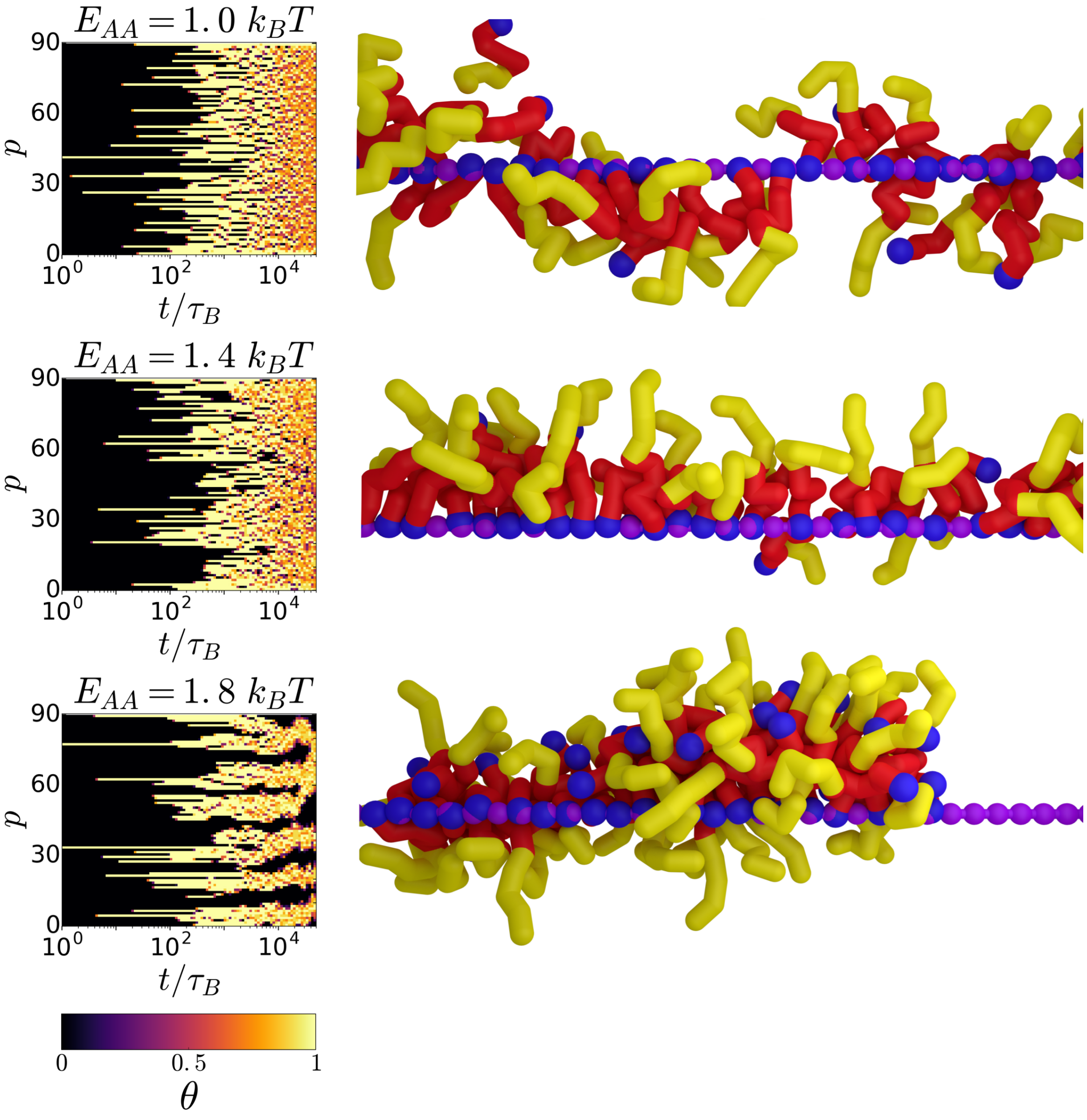

Fig. S2. Template occupancy profiles for simulations with a rigid template. Here we show the occupancy θ of each template domain p over time t/τ_B, for three different values of E_{AA}, highlighting the transition from random Langmuir-like docking at $E_{AA} = 1.0\ k_BT$ to cooperative assembly at $E_{AA} = 1.8\ k_BT$. A snapshot of the final frame of the simulations is shown for each occupancy profile.

(*42*), which is a widely found feature in both experimental template-assembling systems and in theories of the assembly process (*17,22,27,43-45*). With our simulation approach we can thus

reproduce the feature of cooperativity as an emergent property of template-assembling systems. We show how the assembler-assembler interactions provide an important 'dial' for tuning the degree of cooperativity.

Template occupancy profiles simulation details

Scripts for data analysis were written in Python, with the additional modules: Numerical Python (Numpy)(*46*) and Matplotlib (*47*). Simulations were run with $E_{DT} = 17\ k_BT$. For every stored simulation snapshot, we determine whether each template position p is within a distance of $0.5\ \sigma$ from a docking domain, corresponding to the cutoff distance of the U_{DT}. Results were binned into 2000 logarithmic spaced time bins, with the occupancy representing the fraction of snapshots within each bin where a template position was occupied.

Section S3. Overview of all kinetic diagrams

Here, we report the kinetic diagrams and fits over the entire range of the E_{DT} and E_{AA} simulation series. We also show the kinetic diagrams corresponding to the simulations where we introduced allostery.

In figure S4, note the step-wise docking of FA-aggregates that occurs at $E_{AA} \geq 1.2\ k_BT$ in the absence of allostery, that leads to highly stochastic formation of the DA-state. In contrast, the kinetic diagrams for simulations with allostery in figure S5 show a rapid formation of the DA-state for all assembler-assembler interaction strengths above $E_{AA} = 1.0\ k_BT$. Note how the formation of the FA-state is greatly suppressed. Although we do observe small fractions of assemblers in the FA-state here, we show in the next subsection of the SI that these FA-fractions do not correspond to free assembler aggregates, but rather to assemblers that are indirectly attached to the template, via already assembled clusters.

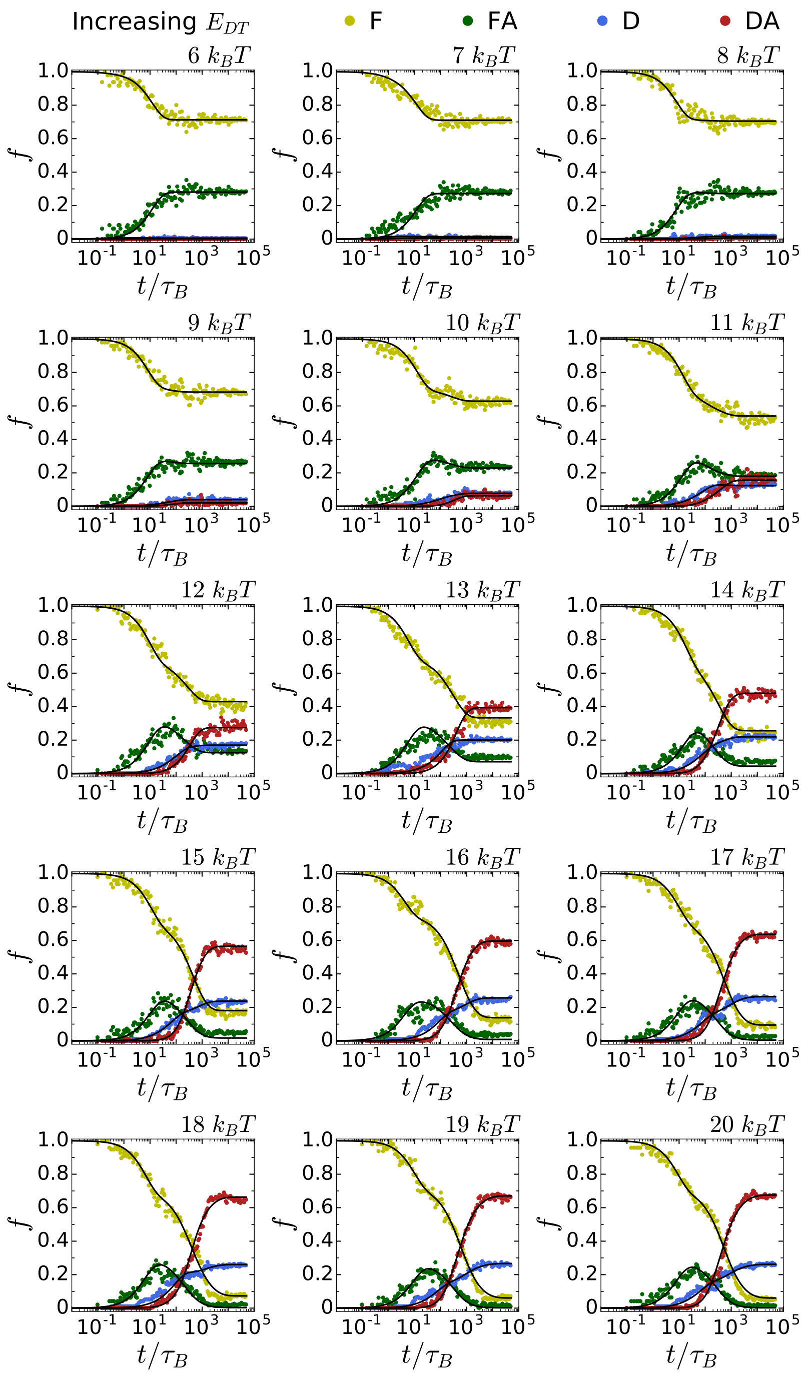

Fig. S3. Overview of kinetic diagrams at varying assembler-template interaction strengths without allostery. Kinetic diagrams are shown for the entire series of simulations at varying assembler-template attraction strengths (E_{DT}) without allostery, showing the fraction of assemblers f in each assembler state over time. Fits of the kinetic model are represented by black lines.

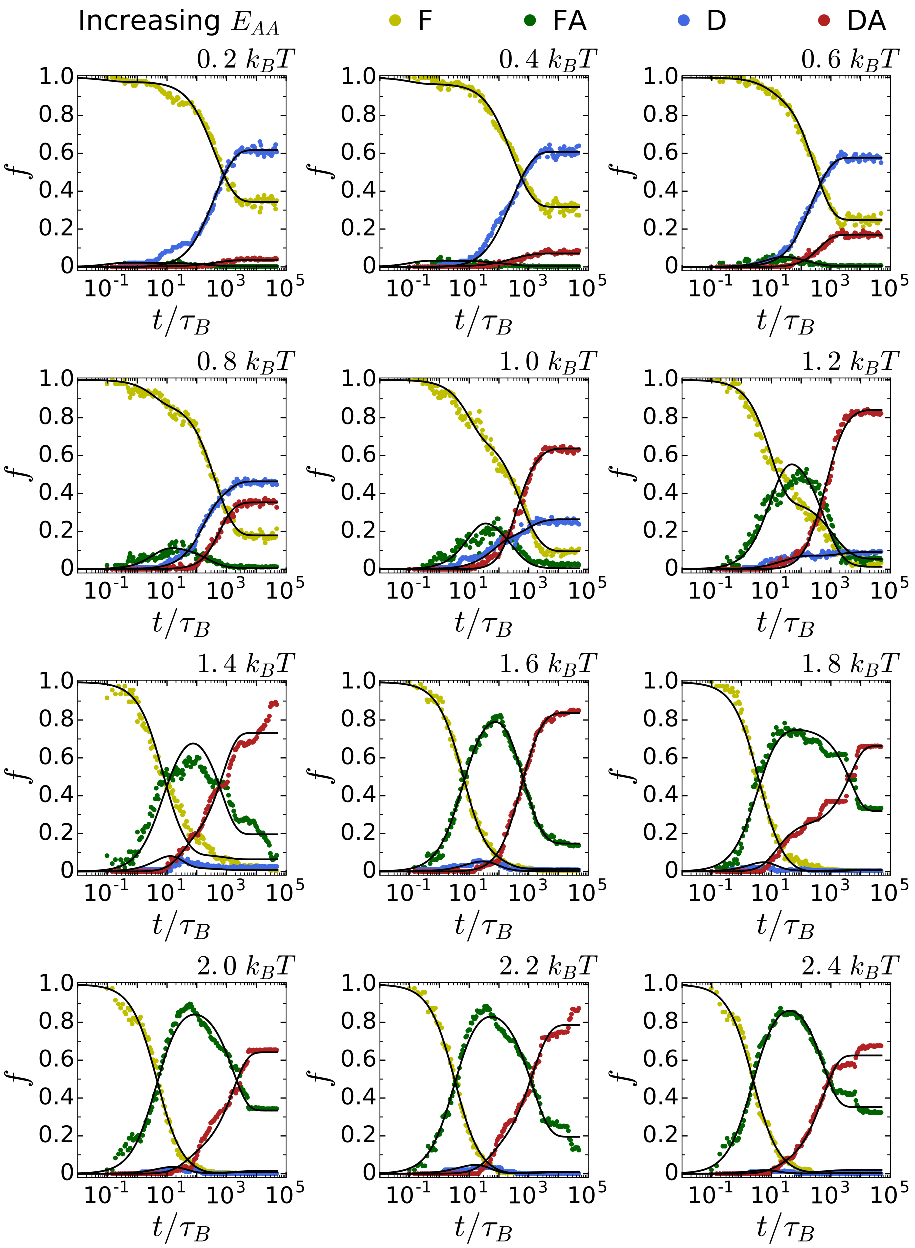

Fig. S4. Overview of kinetic diagrams at varying assembler-assembler interaction strengths without allostery. Kinetic diagrams are shown for the entire series of simulations at varying assembler-assembler attraction strengths (E_{AA}) in the absence of allostery, showing the fraction of assemblers f in each state over time. Fits of the kinetic model are represented as black lines.

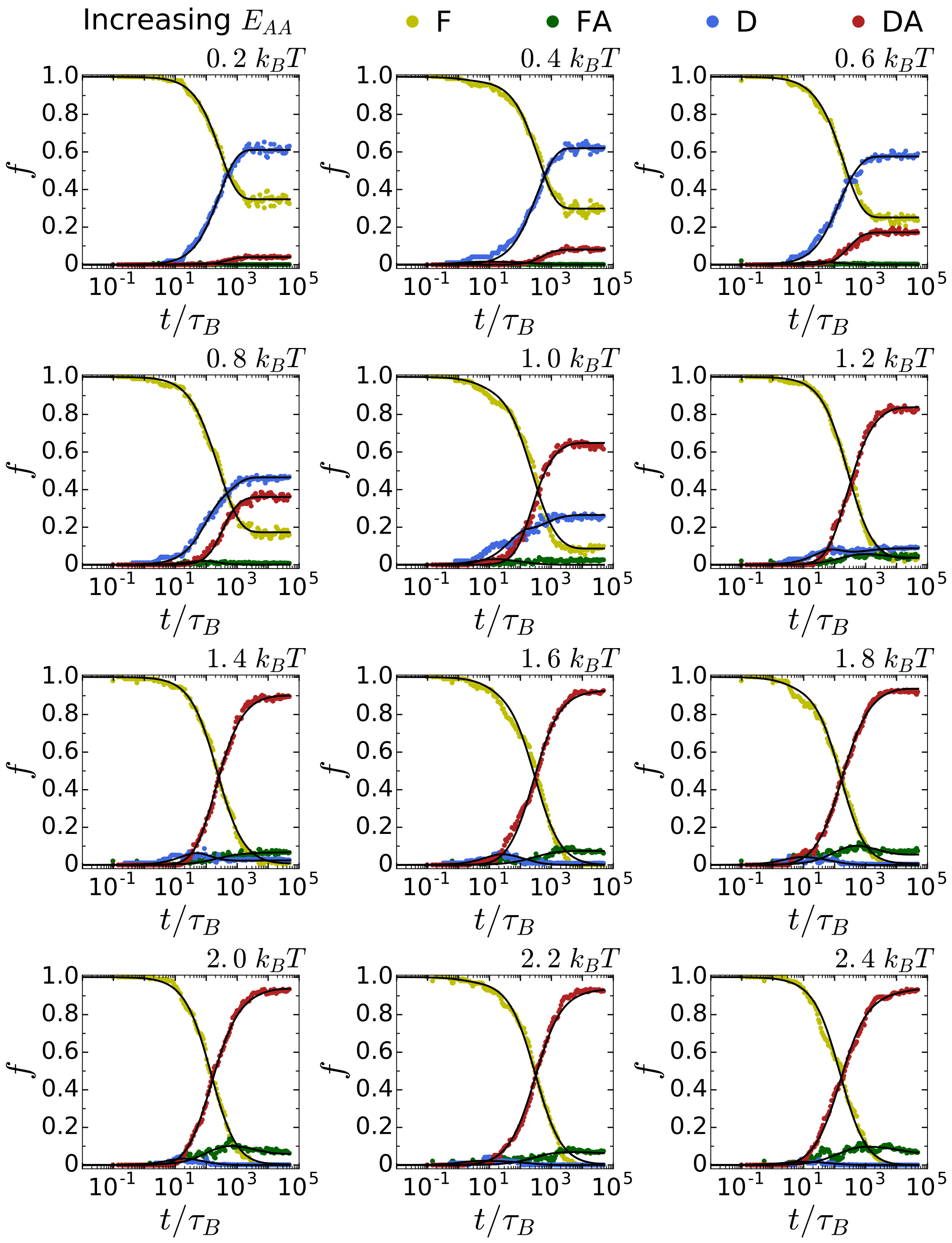

Fig. S5. Overview of kinetic diagrams at varying assembler-assembler interaction strengths including allostery. Kinetic diagrams are shown for the entire series of simulations at varying assembler-assembler attraction strengths (E_{AA}) including allostery, showing the fraction of assemblers f in each state over time.

Section S4. Differentiating between aggregates and recruited assemblers

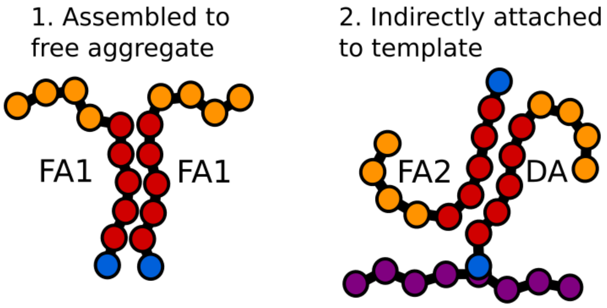

Fig. S6. Schematic representation of two free-assembled (FA) states. A schematic picture is shown of two assembler states, both of which are identified as being in the FA-state. In the first state, FA1, the FA-assembler is part of a free aggregate consisting only of other assemblers in the FA-state, while in the second, FA2, it is indirectly attached to the template, via other assemblers in the DA-state.

In our simulations, the free-assembled (FA) state comprises not only assemblers that are part of aggregates (FA1), but also those that are indirectly attached to the template, via assemblers in the DA-state (FA2, Fig. S6). In this section, we employ a cluster analysis to differentiate between these two states, and discuss the effect of state FA2 on the assembly process, both in the presence and absence of allostery. We show that state FA2 is mostly an infrequent transient state, that predominantly occurs when large aggregates dock onto the template, or as an excess of assemblers when all template positions are occupied.

Our cluster analysis algorithm identifies assemblers that are not docked to the template themselves, but either directly or indirectly attached to the assemblers in the *DA*-state. We label the fraction of these assemblers FA2. All remaining FA-assemblers correspond to freely diffusing aggregates and are labeled as FA1. Using this algorithm, we re-analyze the kinetic diagrams of the simulation series with increasing E_{AA}, both in the presence and absence of allostery. In the absence of allostery (Fig. S7), we generally observe only small fractions of assemblers in

the FA2-state, with respect to the FA1-state. At assembler-assembler attraction strengths of $E_{AA} = 1.4\ k_BT$ or higher, we observe spikes in the FA2 population, that coincide with docking events of large FA1-aggregates. When one of the assemblers is in an aggregate, the remaining assemblers transition from state FA1 to FA2. After each spike the FA2-fraction quickly drops while DA increases, indicating that the remaining assemblers in the aggregate start docking onto the template. This is especially clear for $E_{AA} = 1.8\ k_BT$. In the absence of allostery, state FA2 is therefore mostly an intermediate state that occurs when large aggregates dock onto the template.

If allostery is included in the simulation series (Fig. S8), we no longer observe any assemblers in the FA1-state, again indicating that the formation of aggregates is completely suppressed. As we increase E_{AA} to values greater than $1.0\ k_BT$, we start observing small fractions of FA2-assemblers at the end of the simulations. Our simulations were performed at a small excess of assemblers with respect to template positions. As a result, once all template positions are occupied the remaining assemblers attach to the assembled construct.

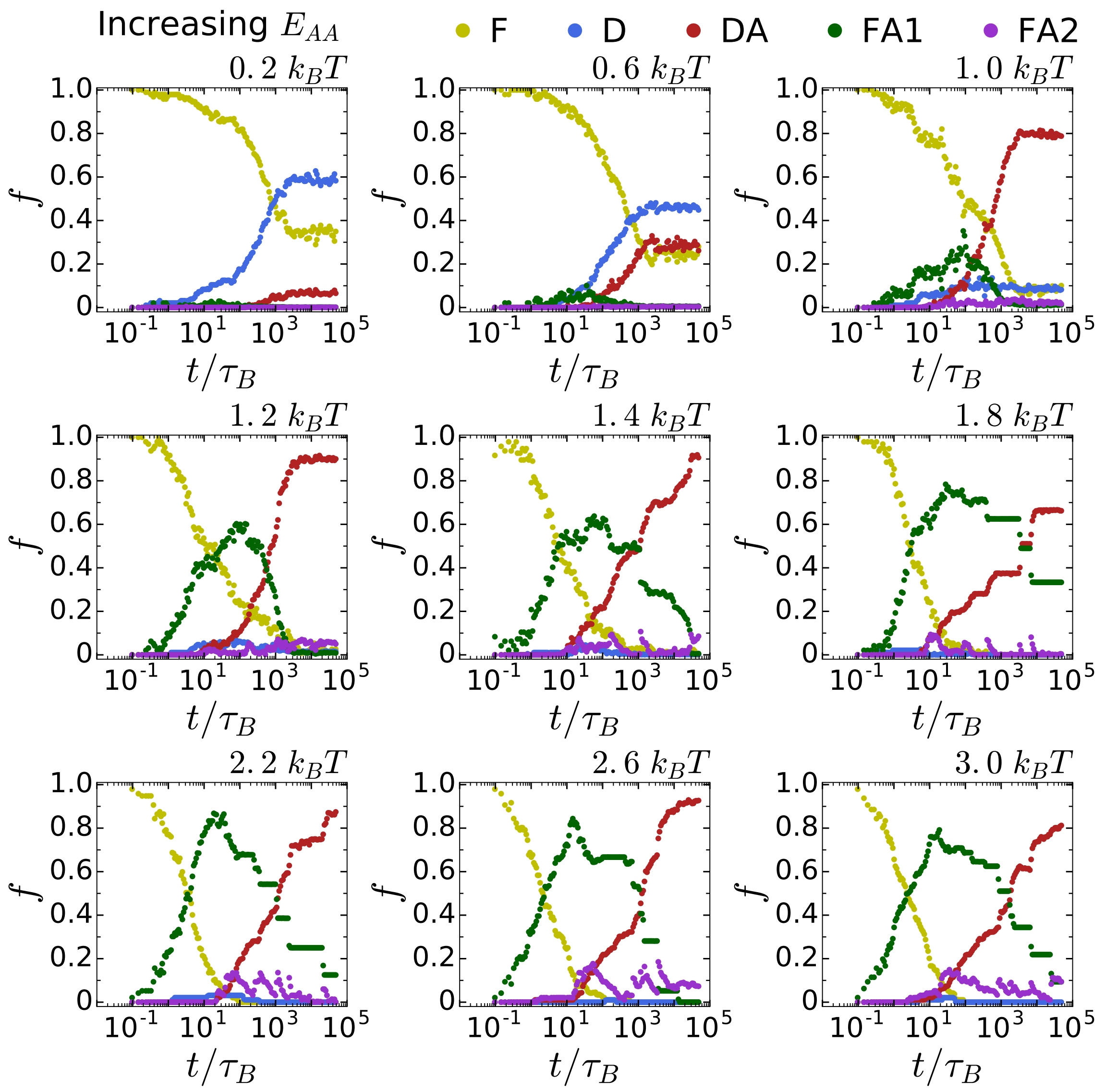

Fig. S7. Overview of kinetic diagrams at varying assembler-assembler interaction strengths without allostery, differentiating between two FA states. Kinetic diagrams are shown for a series of simulations without allostery, at varying assembler-assembler attraction strengths (E_{AA}), showing the fraction of assemblers f in each assembler state over time, including the FA1 and FA2 states.

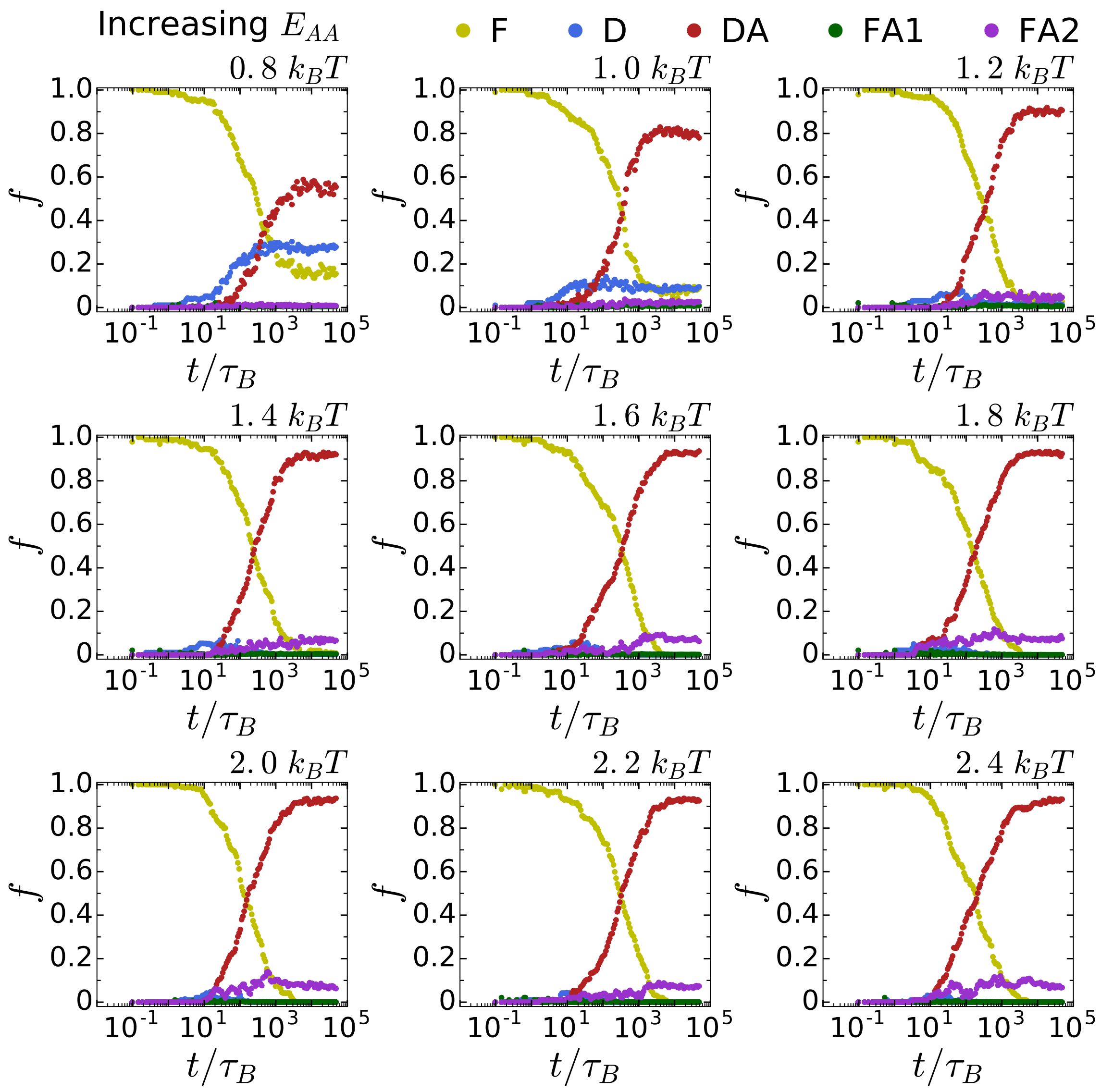

Fig. S8. Overview of kinetic diagrams at varying assembler-assembler interaction strengths including allostery, differentiating between two FA states. Kinetic diagrams are shown for a series of simulations with allostery turned on, at varying assembler-assembler attraction strengths (E_{AA}), showing the fraction of assemblers f in each assembler state over time, including the FA1 and FA2 states.

Section S5. Residuals obtained for the simulated annealing fit algorithm

Our kinetic model contains seven tunable rate constants as fit parameters, which leads to a goodness-of-fit landscape with many local minima. In order to find the global minimum we sample this landscape 100 times with our simulated annealing algorithm, starting from randomized initial values for each of the seven rate constants (see Materials & Methods in the main paper). Here, we report histograms of the residual values obtained by this approach for the E_{DT} and E_{AA} simulation series (Fig. S9 and S10). These histograms show the fit count N for every bin with a sum of squared residuals R. In both figures S9 and S10, we observe a broad distribution of residuals. In each case, we observe ‘spikes’: specific R values with a high count rate, corresponding to local minima where multiple runs of the simulated annealing algorithm end up. The ten fits with the lowest residual values are indicated in red. These were averaged to form the rate constant plots which we discuss in the main paper.

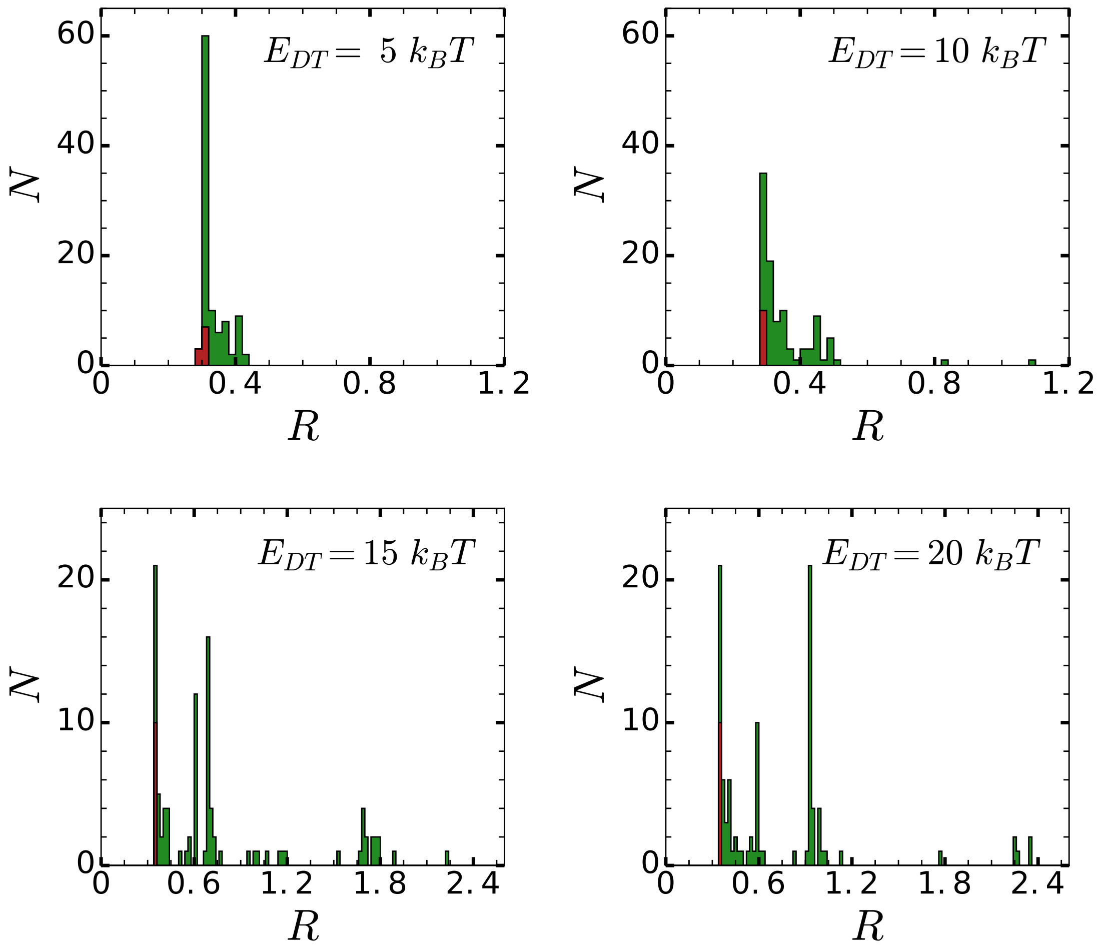

Fig. S9. Histograms of fit residuals at varying assembler-template interaction strength. Histograms are shown of the residuals found by the simulated annealing algorithm, showing the number of fit results (N) with a certain residual value R, as carried out for a series of simulations with varying assembler-template interaction strengths (E_{DT}). The ten fit results with the lowest residual values are indicated in red.

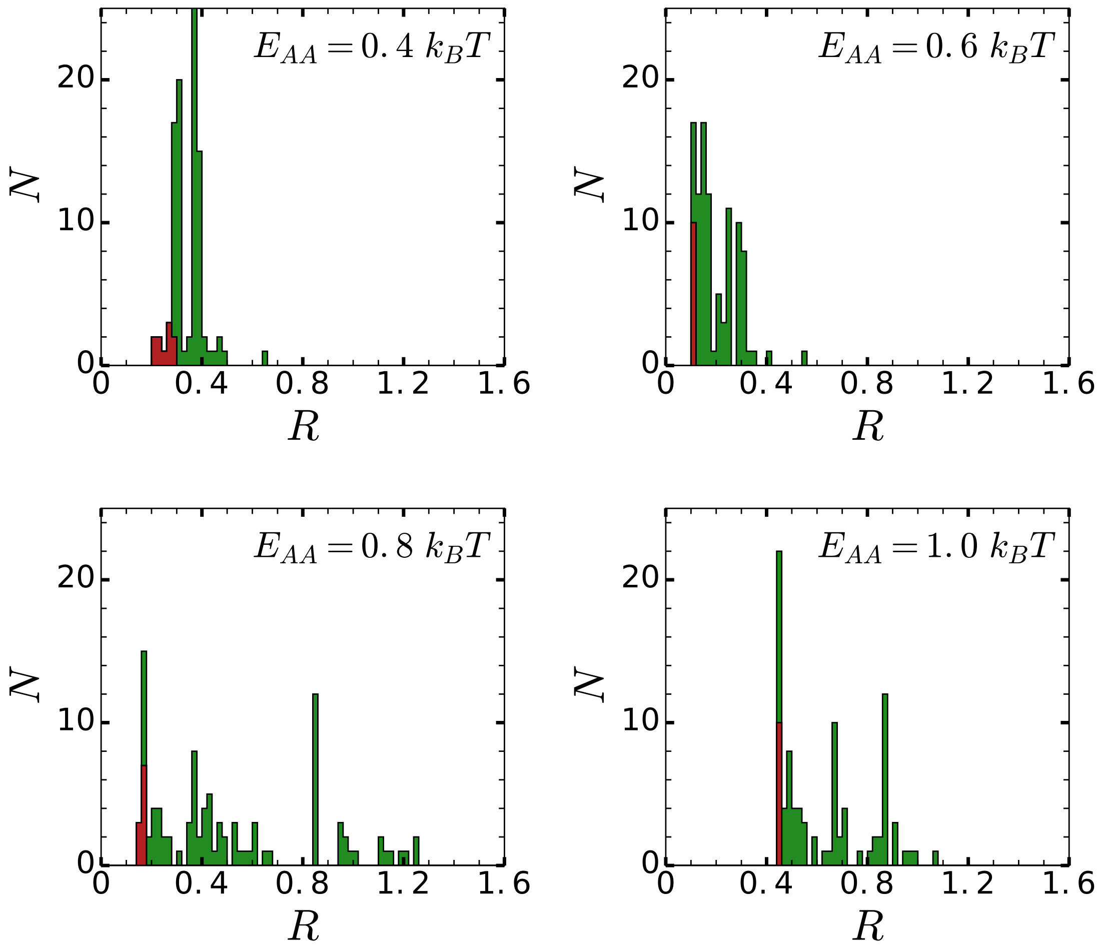

Fig. S10. Histograms of fit residuals at varying assembler-assembler interaction strength. Histograms are shown of the residuals found by the simulated annealing algorithm, showing the number of fit results (N) with a certain residual value R, as carried out for a series of simulations with varying assembler-assembler interaction strengths (E_{AA}). The ten fit results with the lowest residual values are indicated in red.
